# Measurement of Pharyngeal Residue From Lateral View Videofluoroscopic Images

**DOI:** 10.1044/2020_JSLHR-19-00314

**Published:** 2020-05-07

**Authors:** Catriona M. Steele, Melanie Peladeau-Pigeon, Ahmed Nagy, Ashley A. Waito

**Affiliations:** a Swallowing Rehabilitation Research Laboratory, Toronto Rehabilitation Institute, University Health Network, Ontario, Canada; b Department of Speech-Language Pathology, Rehabilitation Sciences Institute, Faculty of Medicine, University of Toronto, Ontario, Canada; c Faculty of Medicine, Fayoum University, Egypt; d Department of Communicative Sciences and Disorders, University at Buffalo, NY

## Abstract

**Purpose:**

The field lacks consensus about preferred metrics for capturing pharyngeal residue on videofluoroscopy. We explored four different methods, namely, the visuoperceptual Eisenhuber scale and three pixel-based methods: (a) residue area divided by vallecular or pyriform sinus spatial housing (“%-Full”), (b) the Normalized Residue Ratio Scale, and (c) residue area divided by a cervical spine scalar (%(C2–4)^2^).

**Method:**

This study involved retrospective analysis of an existing data set of videofluoroscopies performed in 305 adults referred on the basis of suspected dysphagia, who swallowed 15 boluses each (six thin and three each of mildly, moderately, and extremely thick 20% w/v barium). The rest frame at the end of the initial swallow of each bolus was identified. Duplicate measures of pharyngeal residue were made independently by trained raters; interrater reliability was calculated prior to discrepancy resolution. Frequency distributions and descriptive statistics were calculated for all measures. Kendall's τ_b_ tests explored associations between Eisenhuber scale scores and pixel-based measures, that is, %-Full and %(C2–4)^2^. Cross-tabulations compared Eisenhuber scale scores to 25% increments of the %-Full measure. Spearman rank correlations evaluated relationships between the %-Full and %(C2–4)^2^ measures.

**Results:**

Complete data were available for 3,545 boluses: 37% displayed pharyngeal residue (thin, 36%; mildly thick, 41%; moderately thick, 35%; extremely thick, 34%). Eisenhuber scale scores showed modest positive associations with pixel-based measures but inaccurately estimated residue severity when compared to %-Full measures with errors in 20.6% of vallecular ratings and 14.2% of pyriform sinus ratings. Strong correlations (*p* < .001) were seen between the %-Full and %(C2–4)^2^ measures, but the %-Full measures showed inflation when spatial housing area was small.

**Conclusions:**

Generally good correspondence was seen across different methods of measuring pharyngeal residue. Pixel-based measurement using an anatomical reference scalar, for example, (C2–4)^2^ is recommended for valid, reliable, and precise measurement.

Dysphagia is frequently described as involving impairments in two key functional aspects of swallowing, namely, swallowing safety and swallowing efficiency (Clave et al., 2012; Clave & Shaker, 2015). Measures of airway invasion are widely used to describe swallowing safety, and the entry of material into the airway is associated with increased risk for respiratory sequelae (Lakshminarayan et al., 2010; Martino et al., 2005; Pikus et al., 2003; Rofes et al., 2011; Titsworth et al., 2013). Impaired swallowing efficiency, characterized by residue in the pharynx after a swallow, has received less attention (e.g., Molfenter & Steele, 2013; Waito, Tabor-Gray, et al., 2018; Waito et al., 2017). However, the presence of pharyngeal residue has been found to be a risk for subsequent aspiration (Eisenhuber et al., 2002; Molfenter & Steele, 2013), and some studies report an association with malnutrition (Carrion et al., 2015; Clave & Shaker, 2015; Rofes et al., 2010). In order to better understand the links between pharyngeal residue and potential negative sequelae, it is essential that objective measures of residue be employed in research. However, there is currently a lack of consensus regarding preferred metrics for quantifying pharyngeal residue from videofluoroscopy recordings (e.g., Eisenhuber et al., 2002; Han et al., 2001; Hutcheson et al., 2017; Leonard, 2017; Logemann et al., 1989; Martin-Harris et al., 2008; Pearson et al., 2013; Robbins et al., 2007; Rommel et al., 2015; Steele, Mukherjee, et al., 2019; Steele, Peladeau-Pigeon, et al., 2019). Furthermore, thresholds for classifying pharyngeal residue into different degrees of severity, which may have utility in predicting the risk of negative outcomes, are yet to be established or validated. Thus, the definition, prevalence, and implications of residue of concern remain unclear. This technical report compares four different approaches to measuring pharyngeal residue. By applying these measures to an existing data set, we illustrate the different degrees of measurement reliability and precision that are seen and explore trends in the data that reflect concerns regarding validity.

## Background

Table 1 lists several examples of different approaches for rating the severity of pharyngeal residue on lateral view videofluoroscopic images. These approaches can be broadly categorized as follows:

1. visuoperceptual judgments of residue presence (vs. absence) in specific pharyngeal locations,
2. visuoperceptual estimates of residue or bolus clearance as a proportion of the original bolus,
3. visuoperceptual estimates of the degree to which a space (i.e., valleculae or pyriform sinuses) is full of residue, and
4. quantitative pixel-based measurements of residue area.

**Table 1.** Descriptions of different approaches to residue measurement from videofluoroscopy, as described in the literature.

Approach		Parameter	Authors	Description	Scale properties
Visuoperceptual	a. Judgments of residue presence or absence in specific pharyngeal locations	Bolus Residue Scale	Rommel et al. (2015)	1 = *No residue,* 2 = *residue in valleculae*, 3 = *residue on posterior pharyngeal wall or in pyriform sinus*, 4 = *residue in valleculae and on posterior pharyngeal wall or pyriform sinus*, 5 = *residue on posterior pharyngeal wall and in pyriform sinus*, 6 = *residue in valleculae and on posterior pharyngeal wall and in pyriform sinus*	Categorical
	b. Estimates of residue or bolus clearance as a proportion of the original bolus	Oropharyngeal Swallow Efficiency measure	Logemann et al. (1989)	% of Bolus Swallowed / (Oral Transit Time + Pharyngeal Transit Time)	Interval
		Dynamic Imaging Grade of Swallowing Toxicity (DIGEST)	Hutcheson et al. (2017)	Residue measures guiding DIGEST Efficiency Grade: % of Bolus remaining in pharynx after the initial swallow (< 10%, 10%–49%, 50%–90%, > 90%)	Ordinal
		MBS Measurement Tool for Swallow Impairment	Martin-Harris et al. (2008)	Component 16 (Pharyngeal Residue): the amount of bolus material remaining in the pharynx after the first swallow of each discrete bolus: 0 = *absent*, 1 = *trace*, 2 = *a “collection”* (i.e., sufficient to “scoop”), 3 = *> 50% of the original bolus*	Ordinal
	c. Estimates of the degree to which a specific space is full	3-Point ordinal residue scale	Robbins et al. (2007)	Measurements taken in the oral cavity, vallecula, posterior pharyngeal wall, pyriform sinus, and upper esophageal sphincter using a 3-point scale: 0 = *no barium residue*, 1 = *“coating”* (a line of barium on a structure), 2 = *an area of barium larger than a line*	Ordinal
		Functional Dysphagia Scale	Han et al. (2001)	Percent-filled space based on perception of the amount of residue in comparison to the width of the valleculae: 0 = *no residue*, 1 = *< 10% filling*, 2 = *10%–50% filling*, 3 = *50% filling*	Ordinal
		Eisenhuber scale	Eisenhuber et al. (2002)	Percent-filled space based on the perception of the amount of residue in the valleculae or pyriform sinuses in comparison to the height of the space: 0 = *no residue*, 1 = *residue level < 25% of the height of the space*, 2 = *residue level between 25% and 50% of the height of the space*, 3 = *residue level > 50% of the height of the space*	Ordinal
Quantitative pixel based	d. Measures of residue area	%-Full (valleculae; pyriform sinuses)	Steele, Mukherjee, et al. (2019)	(Residue Area / Spatial Housing Area) × 100	Interval
		Normalized Residue Ratio Scale (NRRSv = valleculae, NRRSp = pyriform sinuses)	Pearson et al. (2013)	(%-Full) × [(Residue Area / ((C2–4 Length)^2^) × 10]	Interval
		Bolus clearance ratio	Leonard (2017)	Residue Area / Bolus Area on Frame Prior to UES Opening	Interval
		Pharyngeal residue ratio	Leonard (2017)	Residue Area/Pharyngeal Area at Rest	Interval
		%(C2–4)^2^	Steele, Peladeau-Pigeon, et al. (2019)	Residue Area / ((C2–4 Length)^2^)	Interval

A recent psychometric review concludes that visuoperceptual judgments of pharyngeal residue from videofluoroscopy recordings have reasonable overall quality and reliability (Swan et al., 2019). However, methodological choices that may contribute to variability in these measures include (but are not limited to): the concentration of barium used in the experiment, that is, higher concentrations are more likely to coat the mucosa with the potential to be misidentified as residue (Steele et al., 2013); procedural instructions regarding the selection of frames on which judgments are made (at the end of the initial swallow, the second swallow, etc.; Pearson et al., 2013); operational definitions regarding the amount of residue needed to warrant a decision of “present”; and reference areas or dimensions that are used for scaling judgments of residue severity (see Pearson et al., 2013, for several examples). Pixel-based measures are also vulnerable to these same sources of variability, but they have advantages over visuoperceptual judgments in that measurement rather than estimation should improve precision; similarly, they should be replicable and less prone to poor interrater agreement. Furthermore, pixel-based measures fall on a continuous interval scale, which may be better able to demonstrate small but clinically relevant degrees of change. For example, a recent treatment outcome study (Steele et al., 2016) concluded that tongue pressure resistance training was effective for reducing vallecular residue, measured using the pixel-based Normalized Residue Ratio Scale (NRRS; Pearson et al., 2013), whereas a previous study using a 3-point ordinal scale had failed to detect change (Robbins et al., 2007). One acknowledged limitation of all two-dimensional (2D) lateral videofluoroscopic measures of pharyngeal residue is that they do not properly capture the three-dimensional (3D) nature of residue, including possible asymmetries. Fortunately, a recent comparison between pixel-based area measures on 2D lateral views from 3D computed tomography scans and corresponding volumetric measures has shown a very tight correspondence (*R* ^2^ = .91; Mulheren et al., 2019).

## Objectives

The objective of this analysis was to compare four different approaches to evaluating pharyngeal residue from lateral view videofluoroscopic images:

1. Eisenhuber scale scores (Eisenhuber et al., 2002),
2. %-Full measures (residue area divided by vallecular or pyriform sinus spatial housing; Steele, Mukherjee, et al., 2019),
3. the NRRS (Pearson et al., 2013), and
4. %(C2–4)^2^ measures (residue area divided by a cervical spine scalar; Steele, Peladeau-Pigeon, et al., 2019).

**Figure 1.**
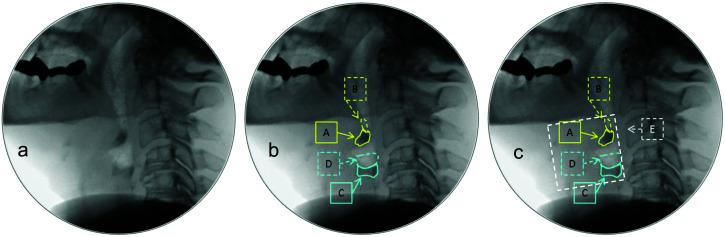
An example image showing pharyngeal residue present in both the valleculae and pyriform sinuses. (a) Residue severity was rated with Eisenhuber scale scores of 3 (valleculae) and 1 (pyriform sinuses). (b) The components required for calculating %-Full measures of residue (i.e., residue area / spatial housing area × 100) are shown as follows: A = vallecular residue area; A + B = vallecular spatial housing area; C = pyriform sinus residue area; C + D = pyriform sinus spatial housing area. In this example, the %-Full measures were calculated as 79.6% full (valleculae) and 6.9% full (pyriform sinuses). (c) The white dashed square (E) illustrates the (C2–4)^2^ reference scalar that is required for additional calculations of residue in %(C2–4)^2^ units or in the equation for the Normalized Residue Ratio Scale (NRRS), that is, [(%-Full) × (%(C2–4)^2^)] / [%(C2–4)^2^ × 10]. In this example, the %(C2–4)^2^ measures were calculated as 2.68% (valleculae) and 1.06% (pyriform sinuses). When these values were plugged into the NRRS equation, the resulting measures were 0.24 (valleculae) and 0.01 (pyriform sinuses).

Figure 1 provides an example image with pharyngeal residue seen in both the valleculae and pyriform sinuses, measured using each of these approaches.

In comparing these different approaches, our specific research questions were as follows:

1. How well do these different measures and their subcomponents perform with respect to interrater reliability?
2. What are the frequency distributions of pharyngeal residue according to these different measures in an example data set?
3. What is the distribution of nonzero Eisenhuber scale scores for the valleculae and pyriform sinuses, relative to the corresponding
  a. %-Full residue measurement scale?
  b. NRRS measurement scale?
  c. %(C2–4)^2^ measurement scale?
4. In cases where nonzero Eisenhuber scale scores do not fall within the expected quartile of the %-Full distribution (e.g., a rating of 1 representing residue filling a space to less than 25% of its height would be expected to have a corresponding %-Full measure of < 25%), what proportion of scores within each Eisenhuber scale level are under- or overestimates?
5. How strongly are the %-Full and %(C2–4)^2^ measures of residue severity correlated?

Strong positive correlations were expected across measurement methods. (Given that both the %-Full and the %(C2–4)^2^ measures are components of the equation for the NRRS, strong relationships with the NRRS can be presumed by definition and were not explored in this study.)

## Method

For the purposes of illustrating similarities and differences across these measures of pharyngeal residue, we used an existing data set of videofluoroscopy recordings from a previously published study (Steele, Mukherjee, et al., 2019). Details regarding the original study methods and results can be found in the published article and its appendix (Steele, Mukherjee, et al., 2019; https://link.springer.com/article/10.1007/s00455-018-09974-5#SupplementaryMaterial).

### Original Study Pharyngeal Residue Measurements

As part of the original study, videofluoroscopy recordings for each bolus were analyzed in duplicate by two trained raters, who were blinded to each other's ratings. Rating was completed according to a standard operating procedure, in which the determination of pharyngeal residue presence and severity involved three steps:

1. identification of the frame of “swallow rest” for each swallow, defined as the first frame showing the pyriform sinuses at their lowest position, relative to the spine, as part of postswallow pharyngeal relaxation prior to onset of a subsequent swallow or nonswallow event;
2. visuoperceptual judgment of residue severity in the valleculae and the pyriform sinuses on each swallow rest frame using the Eisenhuber scale (Eisenhuber et al., 2002); and
3. for cases where residue was judged to be present either in the valleculae and/or the pyriform sinuses (i.e., Eisenhuber scale scores > 0), pixel-based measurements of residue area and spatial housing area on the swallow rest frame, in order to yield %-Full measures for the valleculae and pyriform sinuses.

All pixel-based measures were performed using ImageJ software (https://imagej.nih.gov/ij). Disagreement in Eisenhuber scale scores was operationally defined as any difference of at least one level, and for pixel-based measures, it was defined as any difference greater than 1.6 in the ratio of the absolute difference over the average value of the two provided ratings. Cases demonstrating disagreement according to these criteria were taken to a consensus meeting for remeasurement and resolution. Where rater differences did not require resolution, the smaller (i.e., more conservative) of the two rating values was taken as the rating of record. If the raters concurred that visualization of the structures necessary for a particular rating was obscured, the feature in question was documented as not ratable and became a missing data point. In total, this data set comprised recordings of 3,545 boluses with available residue measures for the valleculae and/or the pyriform sinuses.

### Additional Data Processing for This Technical Report

Comparisons for this technical report were performed using measures from the swallow rest frame at the end of the initial swallow of each bolus. In addition to the measurements made in the initial study, for cases where pharyngeal residue was judged to be present, the length of the C2–4 cervical spine was measured (in pixels) on the initial swallow rest frame. This scalar reference measure enabled calculation of the NRRS and residue in %(C2–4)^2^ units. These measures were derived for the vallecular and pyriform sinus locations separately, and the %(C2–4)^2^ measures were added together for a composite “sum vallecular and pyriform sinus” measure.

### Analyses

Of the 3,545 boluses in the data set, a total of 1,302 (37%) were judged to have residue present (i.e., nonzero Eisenhuber scale scores): 519/1,420 thin boluses (37%), 304/736 mildly thick boluses (41%), 246/701 moderately thick boluses (35%), and 233/688 extremely thick boluses (34%). Interrater reliability was calculated on initial ratings (prior to discrepancy resolution) using Kendall's τ_b_ for the ordinal Eisenhuber scale scores and intraclass correlations for all interval, pixel-based measures. Histograms were inspected to understand frequency distributions, and descriptive statistics were calculated for each continuous parameter (5th, 25th, median, 75th, and 95th percentiles). Comparisons across the different measurement methods were made with the nonzero residue cases only, as follows:

- Eisenhuber scale scores were explored in relation to the pixel-based %-Full, NRRS, and %(C2–4)^2^ measures for the valleculae and pyriform sinuses using cross-tabulations, box plots, and Kendall's τ_b_ tests.
- The accuracy of the Eisenhuber scale ratings for the valleculae and pyriform sinuses was evaluated by cross-tabulation with 25% increments of the %-Full measure (i.e., 1%–25% full, 26%–50% full, > 50% full).
- Scatter plots and Spearman rank correlations were used to explore relationships between the %-Full and %(C2–4)^2^ measures.

## Results

### Interrater Reliability

Table 2 shows interrater reliability for the different measurement methods and their subcomponents. Median and interquartile range values for the observed differences across raters (prior to discrepancy resolution) are also provided. It can be seen that agreement was excellent in the majority of cases. However, pixel-based measures of vallecular and pyriform sinus housing area (which are components in the derivation of %-Full and NRRS measures) showed poorer agreement than the other measures. Vallecular NRRS measures were the only derived measure with an intraclass correlation of < .94.

**Table 2.** Interrater reliability and precision for the different measures of residue and their subcomponents.

Parameter	Method	Measure of agreement	Value	Precision	Interpretation	Median difference	Interquartile range of difference
Swallow rest frame	Judged	Intraclass correlation	.988	95% CI [0.987, 0.989]	Excellent	2 frames	7 frames
Eisenhuber scale score (pyriform sinuses)	Judged	Kendall's τ_b_	.66	*df* = 4610	Strong	0 levels	0 levels
Eisenhuber scale score (valleculae)	Judged	Kendall's τ_b_	.75	*df* = 4459	Strong	0 levels	0 levels
Vallecular residue area (pixels^2^)	Measured	Intraclass correlation	.957	95% CI [0.955, 0.959]	Excellent	0 pixels^2^	3 pixels^2^
Pyriform sinus residue area (pixels^2^)	Measured	Intraclass correlation	.953	95% CI [0.950, 0.955]	Excellent	0 pixels^2^	0 pixels^2^
Vallecular housing area (pixels^2^)	Measured	Intraclass correlation	.931	95% CI [0.923, 0.937]	Good	94 pixels^2^	183 pixels^2^
Pyriform sinus housing area (pixels^2^)	Measured	Intraclass correlation	.635	95% CI [0.591, 0.674]	Moderate	176 pixels^2^	354 pixels^2^
%-Full (valleculae)	Derived	Intraclass correlation	.969	95% CI [0.967, 0.97]	Excellent	0%	0%
%-Full (pyriform Sinuses)	Derived	Intraclass correlation	.972	95% CI [0.970, 0.974]	Excellent	0%	0%
C2–4 length	Measured	Intraclass correlation	.987	95% CI [0.986, 0.988]	Excellent	1.6 pixels	4.3 pixels
NRRSv	Derived	Intraclass correlation	.929	95% CI [0.925, 0.933]	Good	0.00 (no unit)	0.00 (no unit)
NRRSp	Derived	Intraclass correlation	.946	95% CI [0.943, 0.949]	Excellent	0.00 (no unit)	0.00 (no unit)
%(C2–4)^2^–valleculae	Derived	Intraclass correlation	.951	95% CI [0.948, 0.954]	Excellent	0%	0.10%
%(C2–4)^2^–pyriform sinuses	Derived	Intraclass correlation	.95	95% CI [0.947, 0.953]	Excellent	0%	0%
%(C2–4)^2^–sum valleculae and pyriform sinuses	Derived	Intraclass correlation	.956	95% CI [0.954, 0.959]	Excellent	0%	0.30%

*Note.* CI = confidence interval; NRRSv = Normalized Residue Ratio Scale–valleculae; NRRSp = Normalized Residue Ratio Scale–pyriform sinuses.

### Frequency Distributions

The frequencies of different Eisenhuber scale scores for vallecular and pyriform sinus residue are shown by consistency and overall in Table 3. Notably, two thirds or more of the boluses in this data set were judged to have no residue present, regardless of location or consistency. One can also see that vallecular residue was more common than residue in the pyriform sinuses. There is no apparent trend of more frequent residue with thicker consistencies based on Eisenhuber scale scores.

**Table 3.** Frequency distributions for the different Eisenhuber scale scores by consistency and overall.

Location	Eisenhuber scale score	Thin		Mildly thick		Moderately thick		Extremely thick		Overall	
		*n* (boluses)	%	*n* (boluses)	%	*n* (boluses)	%	*n* (boluses)	%	*n* (boluses)	%
Valleculae	0	1005	71	490	67	510	73	498	72	2503	71
	1	281	20	135	18	85	12	94	14	595	17
	2	55	4	56	8	40	6	32	5	183	5
	3	79	6	55	7	66	9	64	9	264	7
Pyriform sinuses	0	1110	78	569	77	576	82	571	83	2826	80
	1	240	17	134	18	102	15	98	14	574	16
	2	42	3	25	3	13	2	10	1	90	3
	3	28	2	8	1	10	1	9	1	55	2

Histograms for all four approaches to residue measurement showed strong positive skews. Table 4 shows percentile descriptive statistics (5th, 25th, median, 75th, and 95th) for the various pixel-based measures for cases with nonzero Eisenhuber scale scores, by consistency and overall. Here, the median, 75th, and 95th percentile values for the vallecular %-Full and NRRS measures show a trend toward greater residue for thicker consistencies. However, the trend is not as apparent using the %(C2–4)^2^ measure, and the opposite trend (i.e., smaller residue values for thicker consistencies) is seen for pyriform sinus and the sum vallecular and pyriform sinus measures in %(C2–4)^2^ units.

**Table 4.** Descriptive statistics (percentiles) for the different residue measures by consistency.

Measure	Location	Consistency	5th percentile	25th percentile	*Mdn*	75th percentile	95th percentile
%-Full	Valleculae	Thin	5.03%	11.25%	19.30%	39.24%	84.63%
		Mildly thick	5.81%	13.58%	23.29%	44.76%	97.54%
		Moderately thick	6.87%	16.15%	30.82%	57.04%	100.00%
		Extremely thick	6.09%	14.90%	30.47%	52.82%	100.00%
		Overall	5.64%	13.13%	24.10%	47.08%	100.00%
	Pyriform sinuses	Thin	3.62%	7.04%	12.06%	21.62%	58.93%
		Mildly thick	2.49%	6.34%	12.33%	21.77%	45.85%
		Moderately thick	2.64%	6.55%	11.33%	21.77%	49.30%
		Extremely thick	2.43%	5.48%	10.92%	21.46%	69.85%
		Overall	2.77%	6.50%	11.66%	21.66%	51.28%
NRRS	Valleculae	Thin	0.002	0.008	0.023	0.077	0.334
		Mildly thick	0.002	0.010	0.035	0.115	0.462
		Moderately thick	0.002	0.014	0.060	0.142	0.652
		Extremely thick	0.002	0.011	0.050	0.146	0.526
		Overall	0.002	0.010	0.033	0.109	0.429
	Pyriform sinuses	Thin	0.001	0.006	0.016	0.058	0.472
		Mildly thick	0.001	0.005	0.018	0.070	0.341
		Moderately thick	0.001	0.004	0.014	0.059	0.279
		Extremely thick	0.001	0.003	0.015	0.050	0.432
		Overall	0.001	0.004	0.016	0.062	0.361
%(C2–4)^2^	Valleculae	Thin	0.28%	0.64%	1.12%	2.01%	5.14%
		Mildly thick	0.25%	0.72%	1.47%	2.58%	6.77%
		Moderately thick	0.24%	0.77%	1.52%	2.84%	7.26%
		Extremely thick	0.22%	0.65%	1.37%	2.88%	6.83%
		Overall	0.25%	0.68%	1.31%	2.43%	6.11%
	Pyriform sinuses	Thin	0.31%	0.84%	1.37%	2.69%	8.97%
		Mildly thick	0.26%	0.70%	1.52%	3.15%	7.24%
		Moderately thick	0.23%	0.62%	1.14%	2.25%	5.69%
		Extremely thick	0.25%	0.48%	1.07%	2.46%	7.09%
		Overall	0.26%	0.70%	1.29%	2.66%	7.07%
	Sum valleculae and pyriform sinuses	Thin	0.32%	0.84%	1.69%	2.99%	9.72%
		Mildly thick	0.28%	0.86%	1.78%	3.62%	9.77%
		Moderately thick	0.32%	0.79%	1.55%	3.20%	10.76%
		Extremely thick	0.28%	0.80%	1.56%	3.16%	8.97%
		Overall	0.30%	0.84%	1.65%	3.15%	9.76%

*Note.* NRRS = Normalized Residue Ratio Scale.

### Comparisons of Eisenhuber Scale Scores to Pixel-Based Measurement Methods

**Figure 2.**
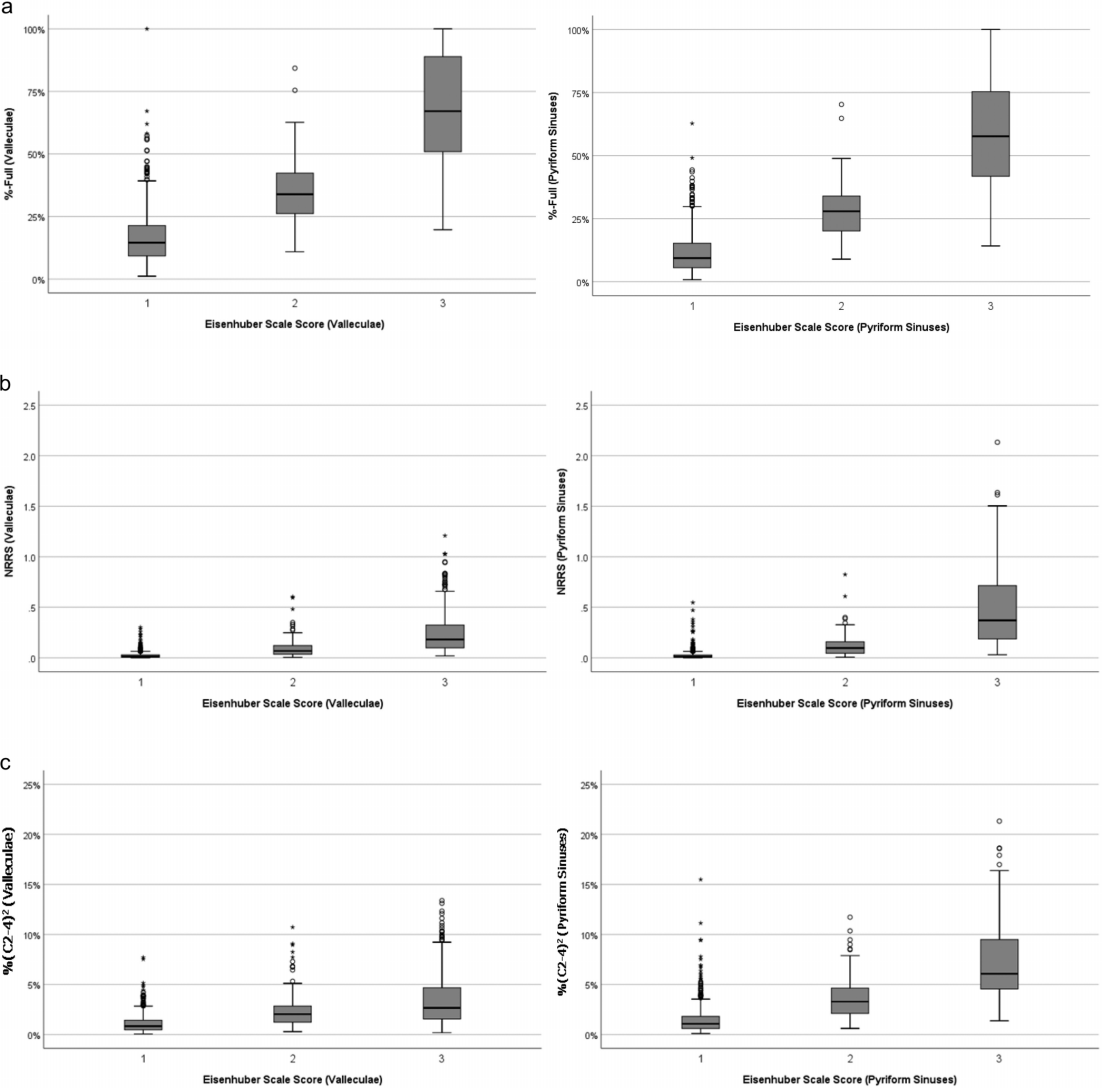
Box plots showing the mapping of Eisenhuber scale scores for the valleculae (left column) and pyriform sinuses (right column) in the data set, according to three different pixel-based approaches to pharyngeal residue measurement: (a) %-Full, (b) Normalized Residue Ratio Scale (NRRS), and (c) %(C2–4)^2^.

Figure 2a illustrates the mapping between nonzero Eisenhuber scale scores and pixel-based %-Full measures of residue in the valleculae and pyriform sinuses, respectively. In both cases, modest to strong positive associations were found between the visuoperceptual ratings and corresponding pixel-based measures (valleculae: τ_b_ = .67, *p <* .001; pyriform sinuses: τ_b_ = .51, *p <* .001). Figure 2b illustrates the mapping between nonzero Eisenhuber scale scores and NRRS measures in the valleculae (left panel) and pyriform sinuses (right panel), respectively. As with the previous comparison, modest to strong positive associations were seen: valleculae, τ_b_ = .61, *p <* .001; pyriform sinuses, τ_b_ = .49, *p <* .001. Similarly, Figure 2c shows the mapping between nonzero Eisenhuber scale scores and residue measures in %(C2–4)^2^ units for the vallecular and pyriform sinuses, respectively. The associations for this comparison were modest: valleculae, τ_b_ = .45, *p <* .001; pyriform sinuses, τ_b_ = .45, *p <* .001.

### Comparison of %-Full and %(C2–4)^2^ measures

**Figure 3.**
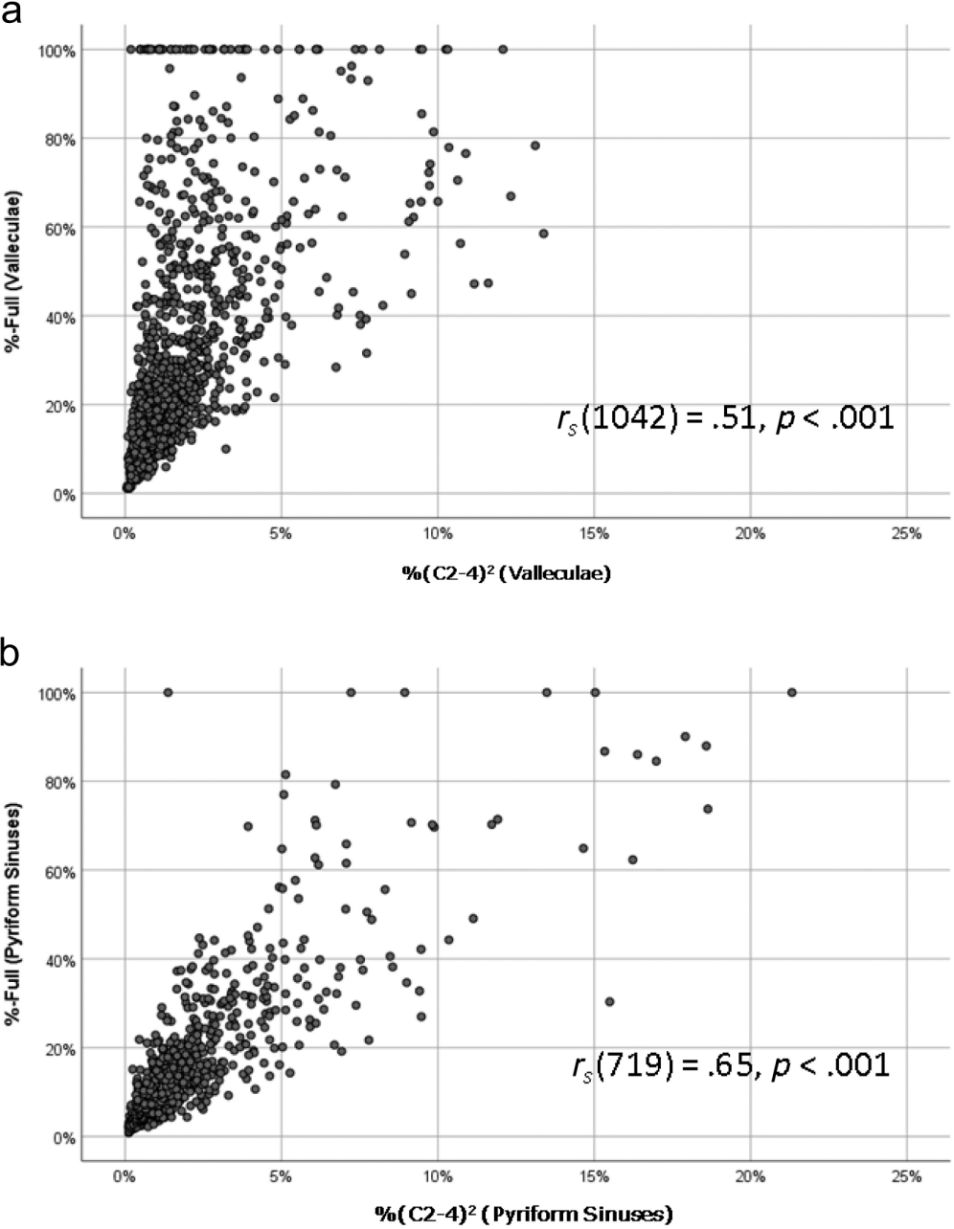
Scatter plots illustrating the correspondence between %-Full and %(C2–4)^2^ measures of pharyngeal residue for (a) the valleculae (top) and (b) the pyriform sinuses (bottom).

Figures 3a and 3b show the relationships between the %-Full and %(C2–4)^2^ measures of residue in the valleculae and pyriform sinuses, respectively. Of note, Figure 3a shows visible clustering in the upper left hand corner of data points with high vallecular %-Full values but small corresponding %(C2–4)^2^ measures. This suggests that the spatial housing area of the valleculae was relatively small on some images, such that a smaller amount of residue filled the available space to a greater degree. Notwithstanding this observation, modest and statistically significant Spearman correlations (*p <* .001) were seen for comparisons of these two different pixel-based measurement approaches.

### Accuracy of Eisenhuber Scale Scores

**Figure 4.**
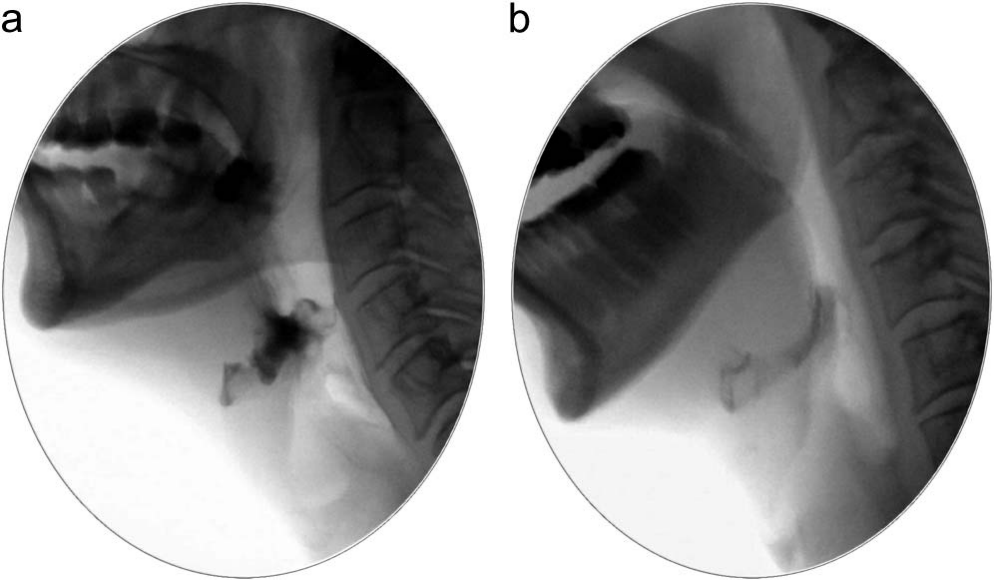
Two contrasting examples of vallecular residue, where the degree to which the vallecular space is collapsed impacts %-Full measures of residue. (a) On the left, the vallecular space was measured to be 87.6% full of residue. (b) On the right, despite the fact that the area of residue in the valleculae appears dramatically lower than in the left-hand image, the vallecular space was measured to be only slightly less full (i.e., 75% full).

When the accuracy of nonzero Eisenhuber scale scores was explored by cross-tabulation with 25% increments of the %-Full measures as reference values, discordant classifications were found for 20.6% of the vallecular ratings and 14.2% of the pyriform sinus ratings. When these were further explored, Eisenhuber scale scores of 1 for vallecular residue (i.e., a residue level of < 25% of the height of the space) were found to be underestimates 16.1% of the time, scores of 2 (i.e., a residue level between 25% and 50% of the height of the space) were found to be underestimates 12.6% of the time and overestimates 21.9% of the time, and scores of 3 (i.e., a residue level of > 50% of the height of the space) were found to be overestimates 21.2% of the time (see Figure 4a). For the pyriform sinuses, Eisenhuber scale scores of 1 were found to be underestimates 8% of the time, scores of 2 were found to be underestimates 2.2% of the time and overestimates 37.8% of the time, and scores of 3 were found to be overestimates 36.4% of the time (see Figure 4b).

## Discussion

In this study, we used a retrospective analysis of an existing data set to illustrate differences between four approaches to measuring pharyngeal residue from lateral view videofluoroscopic images. Several important observations can be gleaned from this study. First, the analysis shows that good interrater agreement can be achieved with all four approaches to measurement. An important caveat to this observation is the fact that the methods in this study began by resolving any differences across raters in selection of the swallow rest frame for the initial swallow of each bolus; this procedural step removed differences in frame selection as a possible source of differences across raters. Although overall interrater agreement appears excellent, the data in Table 2 show that interrater agreement was not as strong for pixel-based measures of spatial housing area. This is a concern, because measures of spatial housing form the denominator for the %-Full measure, and the %-Full measure is also used as a component in calculation of NRRS measures. Evidence that components of these measures may not have good reliability represents a challenge to the apparent reliability of the derived measures.

**Figure 5.**
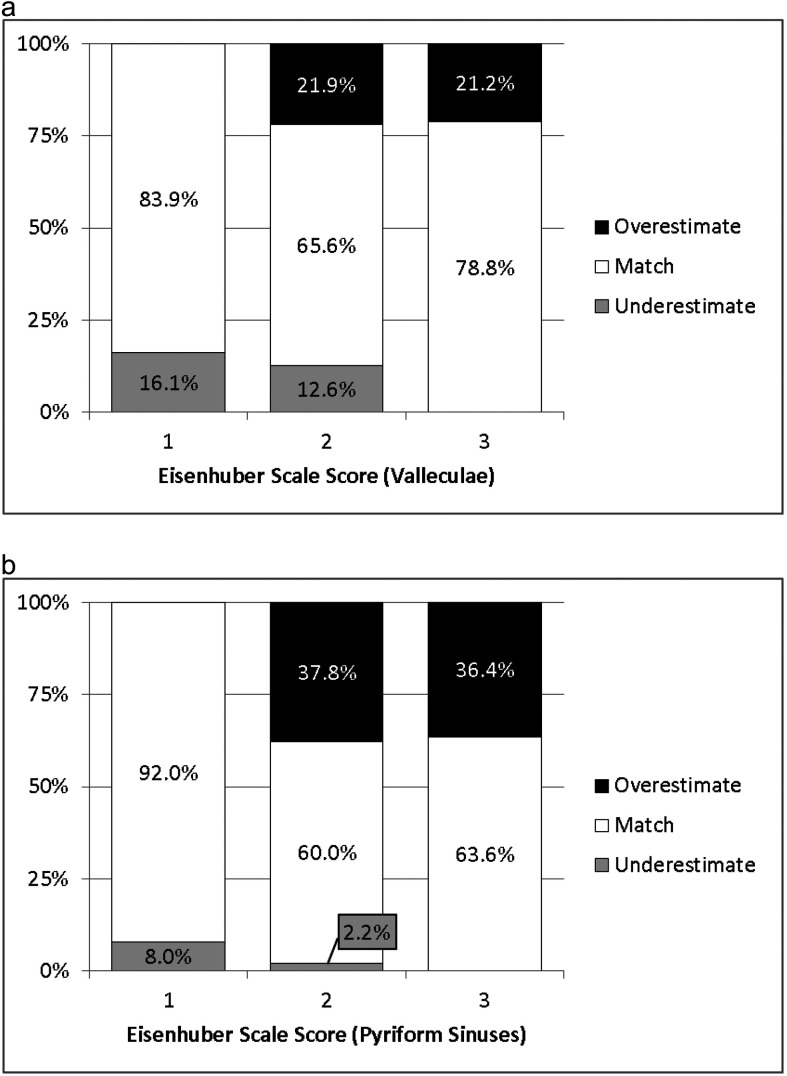
Accuracy of Eisenhuber scale scores when compared to 25% increments of the %-Full measure for (a) valleculae (top) and (b) pyriform sinuses (bottom).

Second, this study raises additional concerns regarding the validity of the %-Full measure, which are apparent in Figure 3a where measures involving the tracing of spatial housing area appear prone to inflating measures of residue severity compared to those using cervical spine reference scalars. The areas of the valleculae and pyriform sinuses may vary as a video recording moves from frame to frame, depending on the position of the epiglottis and the degree of pharyngeal relaxation. The data suggest that %-Full measures may inflate residue severity in cases where spatial housing appears relatively small or collapsed on a lateral view image. Figures 5a and 5b illustrate this issue with two examples of vallecular residue. Additionally, it is acknowledged that the convention used in this study, along with others where spatial housing has been measured (Molfenter & Steele, 2013; Pearson et al., 2013; Steele, Peladeau-Pigeon, et al., 2019; Stokely et al., 2015; Waito, Steele, et al., 2018; Waito, Tabor-Gray, et al., 2018) has been to define the upper boundary of the vallecular spatial housing area using the tip of the epiglottis. In reality, the glosso-epiglottic folds that form the upper lip of the vallecular space are anatomically inferior to this location and are not always easily seen on a lateral view radiographic image. Similarly, it is challenging to know exactly where the upper boundary of the pyriform sinuses lies on a lateral view image.

Third, this study suggests that clinicians are reasonably good at judging degrees of residue severity using visuoperceptual judgments, showing modest associations between Eisenhuber scale scores and corresponding pixel-based measures (see Figures 1a, 1b, and 1c). However, when the accuracy of Eisenhuber scale scores was compared to 25% increments of the %-Full measure, inaccuracies were common, with a trend toward overestimation of residue severity in the visuoperceptual ratings (see Figures 4a and 4b). Given that previous studies also suggest that ordinal scales may lack sensitivity to changes in pharyngeal residue following dysphagia intervention (Robbins et al., 2007), pixel-based methods of measurement are recommended in situations where greater measurement precision is desired, such as pre- versus posttreatment comparisons of residue severity.

For these reasons, we favor the %(C2–4)^2^ measure, which showed excellent interrater reliability for all components and good precision with respect to rater differences (see Table 2). This measure is very similar in construct to the pharyngeal residue ratio proposed by Leonard (2017), in which pixel-based measures of residue area are expressed as a percentage of pharyngeal area at rest. Previous work from our lab suggests that measures of pharyngeal area at rest corresponds to 58% of the (C2–4)^2^ area in healthy adults (Steele, Peladeau-Pigeon, et al., 2019). However, it should be noted that the frames used for measurement of pharyngeal area at rest differ between the Leonard method and our work. Consequently, further studies to confirm the correspondence between the two measures will be needed.

The ability to sum residue measures across different pharyngeal locations for a composite representation of residue severity is an added advantage of the %(C2–4)^2^ approach. In this study, residue measures were only taken from the valleculae and pyriform sinuses; however, residue in other pharyngeal locations, such as coating on the pharyngeal wall, could, in principle, also be measured in %(C2–4)^2^ units and added to the sum vallecular and pyriform sinus measures for a total pharyngeal residue measure (Steele, Peladeau-Pigeon, et al., 2019).

An important observation from the data used in this study is the fact that all measures of residue showed nonnormal distributions with positive skews. This means that comparisons of residue severity should use nonparametric statistics rather than models assuming normality. To date, the field lacks a clear definition of the degree of pharyngeal residue that should be identified as a finding of concern. It is interesting to note that the 75th percentile values for %(C2–4)^2^ measures of residue in the data set used for this study (which comprised adults referred for videofluoroscopy due to suspected dysphagia) are higher than those found in a recently published study in healthy adults under the age of 60 years (Steele, Peladeau-Pigeon, et al., 2019; https://steeleswallowinglab.ca/srrl/wp-content/uploads/ASPEKT-Method-Reference-Value-Tables-V1.3.pdf). It is also interesting to note that the 75th percentile values for the vallecular NRRS measure in this study fall close to the 0.09 cut-point identified by Molfenter and Steele (2013) as representing a risk for penetration–aspiration on a subsequent clearing swallow. Therefore, we propose that the 75th percentile or third quartile boundaries for pharyngeal residue measures in healthy adults represent a meaningful threshold to use as an index of concern in future research exploring the risks associated with pharyngeal residue. The data in this study suggest that vallecular residue is more common than pyriform sinus residue. Therefore, explorations of risk related to residue should include consideration of residue location.

As with any study, this one is not without limitations. It is important to emphasize that the analysis reported in this technical report focused on pharyngeal residue present at the end of the initial swallow for each bolus, such that patterns within individual patients across higher order swallows within boluses or across repeated boluses, either within or across consistencies, have not been taken into consideration in the statistical analyses. Additionally, due to the fact that very limited etiological information was available about participants in the data set, the analysis represents aggregate information for a heterogeneous sample with no history of oncological, structural, or congenital dysphagia but without stratification by diagnosis. Perhaps the most important limitations to note from a clinical perspective are those related to instrumental or research design constraints. All measures of residue severity were taken from 2D lateral view videofluoroscopic images and therefore are unable to capture asymmetries that may exist in the 3D volumetric reality of residue. However, as mentioned earlier, this limitation is somewhat mitigated by findings by Mulheren et al. (2019), who have recently shown tight correspondence between 2D lateral view area measures and 3D volumetric measures of pharyngeal residue.

## Conclusions

In conclusion, this retrospective analysis of pharyngeal residue suggests that visuoperceptual ordinal judgments of residue severity have good interrater reliability and reasonable validity but lack precision. For pixel-based measures that calculate pharyngeal residue relative to measures of the spatial housing area of the valleculae and pyriform sinuses, there are doubts regarding both the validity and reliability of the spatial housing measures. Pixel-based methods employing a stable anatomical reference scalar such as the (C2–4)^2^ area used in this study are recommended for more precise measurement. Further studies will be needed to determine thresholds of pharyngeal residue severity that represent a risk for penetration or aspiration and to evaluate the effectiveness of spontaneous or cued higher order swallows for reducing pharyngeal residue after an initial swallow.

## Author Contributions

Catriona Steele was the principal investigator for the project and was responsible for project design, statistical analysis, and manuscript writing. Melanie Peladeau-Pigeon managed data processing and compiled all the videofluoroscopy ratings for this project, as well as contributing to manuscript editing. Ahmed Nagy and Ashley Waito led the videofluoroscopy rating team for this project and contributed to manuscript editing.
